# Immunization with RBD-P2 and N protects against SARS-CoV-2 in nonhuman primates

**DOI:** 10.1126/sciadv.abg7156

**Published:** 2021-05-28

**Authors:** So-Hee Hong, Hanseul Oh, Yong Wook Park, Hye Won Kwak, Eun Young Oh, Hyo-Jung Park, Kyung Won Kang, Green Kim, Bon-Sang Koo, Eun-Ha Hwang, Seung Ho Baek, Hyeong-Jun Park, Yu-Sun Lee, Yoo-Jin Bang, Jae-Yong Kim, Seo-Hyeon Bae, Su Jeen Lee, Ki-Weon Seo, Hak Kim, Taewoo Kwon, Ji-Hwan Kim, Seonghwan Lee, Eunsom Kim, Yeonhwa Kim, Jae-Hak Park, Sang-In Park, Marta Gonçalves, Byung Mook Weon, Haengdueng Jeong, Ki Taek Nam, Kyung-Ah Hwang, Jihye Kim, Hun Kim, Sang-Myeong Lee, Jung Joo Hong, Jae-Hwan Nam

**Affiliations:** 1 Department of Medical and Biological Sciences, The Catholic University of Korea, Bucheon 14662, Republic of Korea.; 2 National Primate Research Center, Korea Research Institute of Bioscience and Biotechnology, Cheongju, Chungcheongbuk, Republic of Korea.; 3 Department of Research and Development, SK Bioscience, Pangyoro 332, Bundang-gu, Republic of Korea.; 4 Division of Biotechnology, College of Environmental and Bioresources, Jeonbuk National University, Iksan 54596, Republic of Korea.; 5 Department of Laboratory Animal Medicine, College of Veterinary Medicine, Seoul National University, Seoul, Republic of Korea.; 6 Scripps Korea Antibody Institute, Chuncheon, Kangwon-do 24341, Republic of Korea.; 7 Soft Matter Physics Laboratory, School of Advanced Materials Science and Engineering, SKKU Advanced Institute of Nanotechnology (SAINT), Sungkyunkwan University, Suwon 16419, Republic of Korea.; 8 Severance Biomedical Science Institute, Yonsei University College of Medicine, 50-1 Yonsei-ro, Seodaemun-gu, Seoul 03722, Republic of Korea.; 9 Department of Research and Development, SML Genetree, Baumero 225, Seocho-gu, Seoul 06740, Republic of Korea.; 10 Department of Medical Nutrition, Graduate School of East-West Medical Science, Kyung Hee University, Yongin 17104, Republic of Korea.

## Abstract

Since the emergence of severe acute respiratory syndrome coronavirus-2 (SARS-CoV-2), various vaccines are being developed, with most vaccine candidates focusing on the viral spike protein. Here, we developed a previously unknown subunit vaccine comprising the receptor binding domain (RBD) of the spike protein fused with the tetanus toxoid epitope P2 (RBD-P2) and tested its efficacy in rodents and nonhuman primates (NHPs). We also investigated whether the SARS-CoV-2 nucleocapsid protein (N) could increase vaccine efficacy. Immunization with N and RBD-P2 (RBDP2/N) + alum increased T cell responses in mice and neutralizing antibody levels in rats compared with those obtained using RBD-P2 + alum. Furthermore, in NHPs, RBD-P2/N + alum induced slightly faster SARS-CoV-2 clearance than that induced by RBD-P2 + alum, albeit without statistical significance. Our study supports further development of RBD-P2 as a vaccine candidate against SARS-CoV-2. Also, it provides insights regarding the use of N in protein-based vaccines against SARS-CoV-2.

## Teaser

A previously unknown SARS-CoV-2 vaccine protecting nonhuman primates from SARS-CoV-2 challenge was developed using RBD-P2 and N.

## INTRODUCTION

Coronavirus disease 2019 (COVID-19), a disease caused by severe acute respiratory syndrome coronavirus-2 (SARS-CoV-2), is continuously spreading worldwide and has become a serious threat to global health. Therefore, an effective vaccine against SARS-CoV-2 is urgently needed, while only a handful have already been approved. The SARS-CoV-2 genome encodes four structural proteins—spike (S), membrane (M), envelope (E), and nucleocapsid (N) (*1*). Similar to the S protein of SARS-CoV, the S protein of SARS-CoV-2 mediates the entry of the virus into the host cell. The S protein binds to the angiotensin-converting enzyme (ACE) receptor on the host cell through its receptor binding domain (RBD) (*1*, *2*). As neutralizing antibodies against the RBD can block the binding of the virus to host cells, the RBD is considered the main target antigen for vaccines against SARS-CoV-2 (*3*, *4*). Furthermore, RBD-based vaccines are expected to be safer than S protein–based vaccines, as they are not likely to induce T helper 2 (T_H_2)–type immunopathology through antibody-dependent enhancement (*5*–*7*).

The N proteins of coronaviruses are highly immunogenic and are abundantly expressed throughout the course of the infection (*8*). According to a recent study, antibodies against the N protein are more sensitive indicators than anti–S protein antibodies for the detection of early SARS-CoV-2 infection (*9*). Moreover, the N protein of SARS-CoV-2 is believed to induce T cell responses. An adenoviral vector encoding the N protein induced T cell responses in rhesus monkeys, and mice vaccinated with the N protein exhibited T cell proliferation and cytotoxic T cell responses (*10*, *11*). However, in vivo protection conferred by vaccines containing the N protein has not yet been experimentally validated. Thus, it is still ambiguous whether immunization with the N protein combined with RBD would manifest better or worse protective effects.

Currently, numerous SARS-CoV-2 vaccines based on different platforms are being developed owing to urgent demand and importance (*12*). Subunit vaccines are the most commonly used vaccines owing to their safety parameters. Many commercially approved vaccines against diseases caused by viruses such as human papillomavirus, hepatitis B, influenza, and varicella zoster are being manufactured using this platform (*13*). However, because of their lower immunogenicity compared with that of live attenuated vaccines, subunit vaccines need adjuvants, carriers, or engineering of the proteins to boost their immunogenicity (*14*).

Here, we developed a previously unidentified SARS-CoV-2 subunit vaccine (RBD-P2) by fusing the P2 epitope of tetanus toxoid with the RBD of the spike protein to increase its immunogenicity. A previous study shows that the inclusion of the universal toxoid CD4^+^ T cell epitope P2 substantially increases the immunogenicity of a recombinant rotavirus subunit vaccine (*15*). Furthermore, we tested the efficacy of RBD-P2 with or without the N protein in mice, rats, and nonhuman primates (NHPs).

## RESULTS

### Characterization of recombinant RBD-P2 and N

**Fig. 1:**
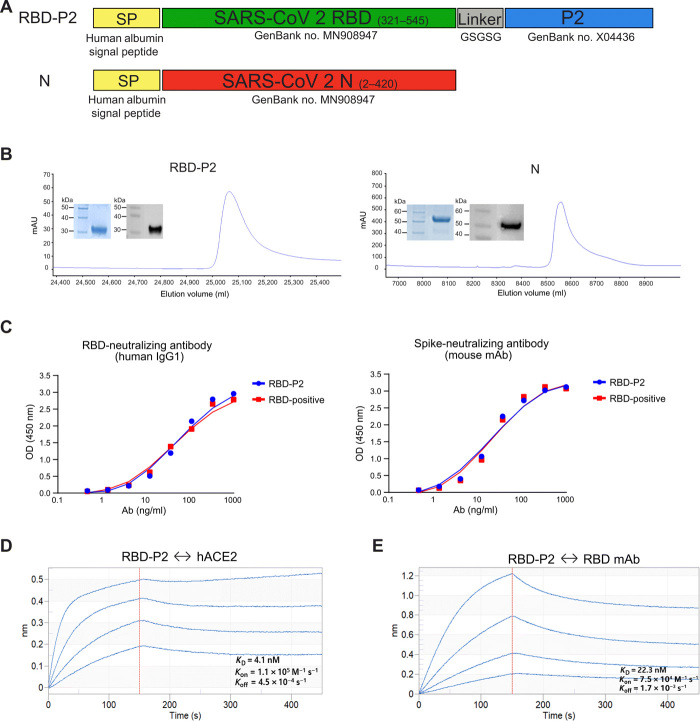
Characterization of RBD-P2 and N. (**A**) Schematic view of RBD-P2 and N. Different domains and elements are marked, including the signal peptide (SP), RBD, linker, and P2, and the relevant GenBank accession numbers are indicated. (**B**) Representative elution chromatograph of the recombinant RBD-P2 and N proteins using three columns (anion exchange, hydrophobic interaction, and multifunctional resin). Inset figures show the SDS–polyacrylamide gel electrophoresis (blue) and Western blot (gray) analyses of the eluted proteins. (**C**) ELISA binding curves of human SARS-CoV-2–neutralizing antibodies (Ab) to RBD and anti-spike mouse monoclonal antibodies (mAb) to RBD-P2. RBD-positive is a commercially procured RBD protein (Sino Biological, catalog no. 40592-V08B). OD, optical density. (**D**) Real-time binding profile of purified RBD-P2 protein to hACE2 and anti-RBD monoclonal antibodies obtained using an Octet system.

We designed and synthesized RBD fused with the P2 epitope of tetanus toxoid (RBD-P2) as a vaccine candidate against SARS-CoV-2. The RBD-P2 and N proteins of SARS-CoV-2 were expressed and purified using High Five insect cells and the baculovirus-mediated Bac-to-Bac expression system. A human albumin signal peptide sequence was fused with the RBD-P2 and N protein sequences to enable the secretion of the proteins (Fig. 1A and fig. S1). Recombinant RBD-P2 and N proteins were successfully purified from the culture supernatant (Fig. 1B). The purity of the final protein preparation was approximately 90%, and the molecular weights of the purified RBD-P2 and N proteins were approximately 32 and 53 kDa, respectively (Fig. 1B). Both proteins were presumably glycosylated as their estimated molecular weights were higher than the theoretical molecular weights calculated on the basis of the amino acid sequences. The binding ability of RBD-P2 was measured using a functional enzyme-linked immunosorbent assay (ELISA). Immobilized RBD-P2 (2 μg/ml) bound to human anti–SARS-CoV-2 RBD-neutralizing antibody (ACROBiosystems, catalog no. SAD-S35) and mouse anti–SARS-CoV-2 spike-neutralizing antibody (Sino Biological, catalog no. MM57) (Fig. 1C). The median effective concentration (EC_50_) of RBD-P2 for the human antibody was 56.5 μg/ml, and that for the murine antibody was 20 μg/ml. These values were similar to those of RBD-positive protein (Sino Biological, catalog no. 40592-V08B; EC_50_: 49.4 μg/ml for human antibody and EC_50_: 22.3 μg/ml for murine antibody). The binding affinity of RBD-P2 for human ACE2 (hACE2) was calculated as follows: *K*_D_ (equilibrium constant) = 4.1 nM with *K*_on_ = 1.1 × 10^5^ M^−1^_S_^−1^ and *K*_off_ = 4.5 × 10^−4^ s^−1^ (Fig. 1D). The binding affinity of RBD-P2 for anti-RBD monoclonal antibody was calculated as follows: *K*_D_ = 22.3 nM with *K*_on_ = 7.5 × 10^4^ M^−1^_S_^−1^ and *K*_off_ = 1.7 × 10^−3^ s^−1^ (Fig. 1E).

### Analysis of humoral and cellular immune responses in mice immunized with RBD-P2 or RBD-P2/N

In this study, we chose alum as an adjuvant because of its strong safety record (*16*). First, we compared the immune responses of RBD-P2 and RBD. RBD was expressed and purified from insect cells using the Bac-to-Bac expression system as described for RBD-P2 (details are in Materials and Methods). After immunization in rats, RBD-P2 + alum induced a significantly higher neutralizing antibody titer than RBD + alum (fig. S2B). Therefore, all vaccine tests were conducted using RBD-P2. We confirmed the complex formation of RBD-P2 and RBD-P2/N with alum by using holotomography and dynamic light scattering (fig. S3A). As shown in fig. S3A, unlike alum alone, which had more scattered clusters, both RBD-P2 and RBD-P2/N with alum clustered in long rod-like shapes. We also used dynamic light scattering analysis to confirm the successful formulation of RBD-P2 and RBD-P2/N with alum. We could detect one main peak in both RBD-P2 + alum and RBD-P2/N + alum. This result indicates that most of the proteins formed a complex with alum (fig. S3B).

To evaluate the stability of alum/protein (RBD-P2 or RBD-P2/N) complexes, we stored alum-adjuvanted RBD-P2 or RBD-P2/N at 4° to 8° or 37°C for 3 months. Alum-formulated samples were centrifuged at different time points, and the supernatants were discarded. Concentrations of remaining protein were measured using the Bicinchoninic acid (BCA) assay. On the basis of the results, both RBD-P2 and RBD-P2/N formulated with alum maintained about 95% stability at least until 3 months at 4° to 8°C, and about 63% stability at 37°C for at least 7 days. This means that formulation of RBD-P2 or RBD-P2/N with alum is very stable at the proper temperature.

**Fig. 2:**
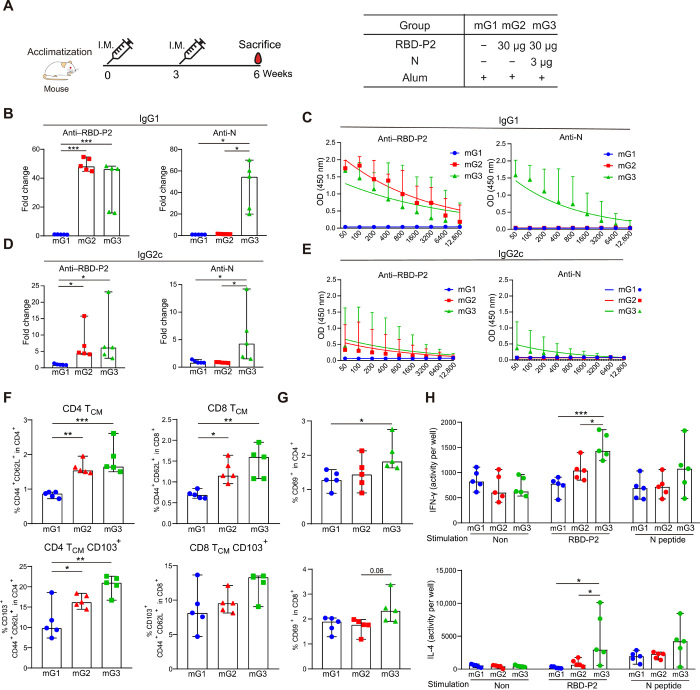
Humoral and cellular immune responses induced by RBD-P2 or RBD-P2/N in mice. (**A**) Overall study design. Mice were intramuscularly immunized with RBD-P2 (30 μg per mouse) + alum or RBD-P2 (30 μg per mouse)/N (3 μg per mouse) + alum at 0 and 3 weeks and euthanized at 6 weeks. (**B** and **D**) RBD-P2– and N-specific IgG1 and IgG2c levels were measured by ELISA at the time of sacrifice (*n* = 5). The fold change represents an increase in the test group (mG2 and mG3) proportional to the average OD_450_ value of the control group (mG1). (**C** and **E**) Sera were tested at different dilutions to detect IgG1 and IgG2c against RBD-P2 and N (*n* = 5). (F to H) Splenocytes (5 × 10^5^) from each mouse were cultured in the presence of RBD-P2 and peptide mix (table S1) together or separately for 2 days (*n* = 5). (**F** and **G**) Frequency of T_CM_ and expression of CD103 and CD69 in T cells analyzed using flow cytometry (*n* = 5). (**H**) Number of IFN-γ– and IL-4–producing cells and the activities of stained cells estimated using the AID EliSpot Reader (*n* = 5 mice). *P < 0.05, **P < 0.01, and ***P < 0.001.

To test the potential of RBD-P2 as a SARS-CoV-2 vaccine candidate, mice were intramuscularly immunized twice with 30 μg of RBD-P2 + alum (mG2) 3 weeks apart. To confirm the effectiveness of the N protein, mice were immunized with 30 μg of RBD-P2 and 3 μg of N + alum (mG3), designated as RBD-P2/N. Mice immunized twice with alum without antigen were designated as mG1. All mice were euthanized 3 weeks after the booster dose (Fig. 2A).

To assess humoral responses, serum samples were collected at the time of euthanization, and RBD-P2– and N-specific immunoglobulin G1 (IgG1) and IgG2c antibody levels were analyzed using ELISA. As shown in Fig. 2 (B to E), RBD-P2 + alum–immunized mice exhibited induction of IgG1 and IgG2c against RBD-P2. The fusion of N with RBD-P2 (RBD-P2/N) slightly reduced the levels of RBD-specific IgG1 but not IgG2c (Fig. 2, B to E). Understandably, N-specific antibodies were detected only in mG3.

Next, to determine cell-mediated immune responses, splenocytes from immunized mice were stimulated with a mix of SARS-CoV-2 RBD-P2 and N peptide mix (table S1) for 2 days to detect antigen-specific T cell responses. As shown in Fig. 2 (F to H), the percentages of both CD4^+^ and CD8^+^ CD44^hi^CD62L^hi^ central memory type T cells (T_CM_) were increased in mG2 and mG3 compared with those in mG1. The expression of CD103, a marker of tissue-residential memory T cells, was increased in the CD4^+^ T_CM_ of mG2 and mG3 (Fig. 2F). The expression of CD69, a T cell activation marker, was increased only in CD4^+^ T cells of mG3 and not in mG1and mG2 (Fig. 2G). To further evaluate the T_H_1/2 balance, interferon-γ (IFN-γ)– and interleukin-4 (IL-4)–producing cells were measured by enzyme-linked immunosorbent spot (ELISpot) assays. Notably, IFN-γ– and IL-4–producing cells were increased in mG3 compared with their numbers in mG2, even when splenocytes were stimulated with RBD alone (Fig. 2H). To test whether immunization with the N protein could induce protective immunity, hACE2 mice were intramuscularly immunized twice with 30 μg of the N protein with alum at 2-week intervals and challenged with SARS-CoV-2 1 week after the second immunization (fig. S4A). Viral titers in the lungs were reduced in N protein–immunized mice, and parenchymal inflammation scores of mG2 were slightly reduced compared with those of mG1. Thus, immunization with N protein + alum could induce a certain degree of protective effect against SARS-CoV-2 (fig. S4, B to E).

### Additional immunization with the N protein increased the levels of neutralizing antibodies in rats

**Fig. 3:**
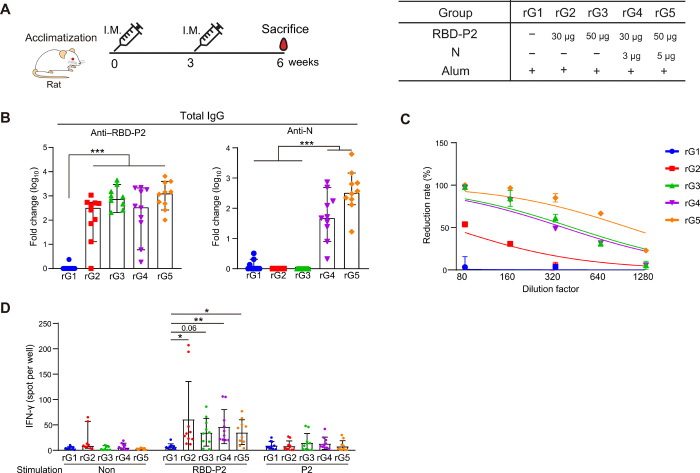
Humoral and cellular immune responses elicited by RBD-P2 or RBD-P2/N in rats. (**A**) Overall study design. Rats were intramuscularly immunized with RBD-P2 (30 or 50 μg per rat) + alum or RBD-P2 (30 or 50 μg per rat)/N (3 or 5 μg per rat) + alum at 0 and 3 weeks and euthanized 6 weeks after immunization (*n* = 10). (**B**) RBD-P2– or N-specific total IgG levels were measured using ELISA at the time of euthanization. (**C**) SARS-CoV-2–neutralizing activity (reduction rate) of serum from immunized rats was determined using the standard plaque reduction neutralization test (PRNT_50_) 6 weeks after the first immunization. (**D**) Splenocytes (5 × 10^5^) from each rat were seeded and stimulated with RBD-P2 or P2 peptides (table S1) for 60 hours before measuring the number of IFN-γ–producing cells. *P < 0.05, **P < 0.01, and ***P < 0.001.

To further assess the immune responses elicited by RBD-P2 or RBD-P2/N, rats were injected with 30 or 50 μg of RBD-P2 (rG2 and rG3) or in combination with N at a ratio of 10:1 (rG4 and rG5) together with alum. The immunization was performed twice (with a 3-week interval in between), and the rats were euthanized 6 weeks after the first injection (Fig. 3A). Both anti–RBD-P2 IgG and anti-N IgG were detected 6 weeks after the first immunization. Immunization with 50 μg of RBD-P2 + alum induced slightly higher levels of RBD-P2–specific antibodies than that induced by 30 μg RBD-P2 + alum, although the difference was not statistically significant (Fig. 3B). RBD-P2/N (rG4 and rG5) + alum immunization yielded an approximately 20 to 50% increase in reduction rates compared with those achieved using each of its RBD-P2 counterparts (rG2 and rG3, respectively) (Fig. 3C). The number of IFN-γ–producing cells in the splenocytes of immunized rats increased in all immunized groups (rG2 to rG5) compared with that observed in rG1 after stimulation with RBD-P2, the peptide mix, or the P2 peptide (table S1). However, no significant differences were observed between RBD-P2/N and RBD-P2 groups (Fig. 3D).

To assess the immunogenicity and toxicity of RBD-P2 or RBD-P2/N in rats, the animals were immunized three times at 2-week intervals with RBD-P2 + alum (rG2) or RBD-P2/N + alum (rG3) according to the guidelines for SARS-CoV-2 vaccine development established by the Korean Food and Drug Administration (fig. S5A). In this experiment, rG3 produced higher levels of anti–RBD-P2 IgG than rG2 at 2 and 4 weeks after the first immunization in females (fig. S5B). However, 6 and 8 weeks after immunization, the levels of anti–RBD-P2 IgG produced in response to immunization with rG2 were markedly increased and reached levels similar to those of rG3. In addition, biochemical analysis revealed that neither RBD-P2 + alum nor RBD-P2/N + alum induced significant toxicity (fig. S5C).

### NHPs immunized with RBD-P2 or RBD-P2/N were protected from SARS-CoV-2 challenge

**Fig. 4:**
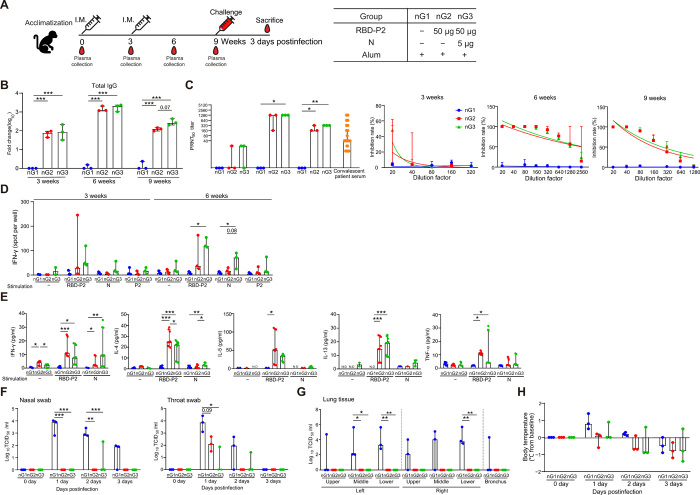
Immunization with RBD-P2 or RBD-P2/N protected NHPs from SARS-CoV-2 challenge. (**A**) Overall study design. NHPs were intramuscularly immunized with alum-adjuvanted RBD-P2 (50 μg) or RBD-P2 (50 μg)/N (5 μg) at 0 and 3 weeks and challenged with SARS-CoV-2 at 9 weeks after the first immunization. All NHPs were euthanized 3 days after the challenge (*n* = 3). (**B**) Fold change in IgG levels of NHPs measured by ELISA at 3, 6, and 9 weeks. (**C**) SARS-CoV-2–neutralizing activity of plasma from immunized NHPs at 3, 6, and 9 weeks after the first immunization, and convalescent patients were analyzed using the standard PRNT_50_. NHPs: *n* = 3; patients: *n* = 31. (**D**) 3 and 6 weeks after immunization, PBMCs were isolated from immunized NHPs and stimulated with RBD-P2, N, or P2 peptides (table S1) for 60 hours before measuring the number of IFN-γ–producing cells. (**E**) PBMCs were isolated from immunized NHPs 6 weeks after immunization and stimulated with RBD-P2 or N for 3 days. Cytokine concentrations were measured in cell culture supernatants using multiplex analysis. (**F** and **G**) Nonvaccinated (nG1), RBD-P2 + alum–vaccinated (nG2), and RBD-P2/N + alum–vaccinated (nG3) NHPs were challenged with SARS-CoV-2 at the TCID_50_ of 2.6 × 10^6^. The challenge was provided through intratracheal, oral, conjunctival, and intranasal routes at 9 weeks after the first immunization. Samples were collected from nasal swabs, throat swabs, and lung tissue at multiple time points after the challenge, and live virus titers were determined by calculating TCID_50_ values. (**H**) Body temperature of NHPs after the challenge. *P < 0.05, **P < 0.01, and ***P < 0.001.

Last, we tested the efficacy of our vaccine candidates in NHPs. Three NHPs per group were intramuscularly administered with either RBD-P2 + alum (nG2) or RBD-P2/N + alum (nG3) at 0 and 3 weeks. Control NHPs (nG1) were administered only with alum adjuvant (Fig. 4A). Three weeks after immunization, the levels of total IgG were increased approximately 100-fold in nG2 and nG3, and at 6 weeks, an approximately 2000- to 3000-fold increase in total IgG levels was detected in both nG2 and nG3. At 9 weeks, total IgG levels were reduced compared to those at 6 weeks. Total IgG levels of nG3 were slightly higher than those of nG2 (Fig. 4B). Neutralizing antibody responses against SARS-CoV-2 were markedly increased in nG2 and nG3 at 6 weeks; however, they were reduced at 9 weeks in both nG2 and nG3. Although additional immunization with N increased the neutralizing antibody titers in NHPs, the increase was not statistically significant (Fig. 4C). Notably, the neutralizing antibody titers induced by RBD-P2 or RBD-P2/N were significantly higher than those of a panel of serum samples from convalescent patients [at 6 weeks, the geometric mean titer (GMT) of nG2 = 507.96 and the GMT of nG3 = 1015.93; at 9 weeks, the GMT of nG2 = 163, the GMT of nG3 = 320, and the overall convalescent serum of GMT = 109.4; all GMT values were calculated on the basis of plaque reduction neutralization test (PRNT_50_) values] (Fig. 4C).

To assess T cell responses, the number of IFN-γ–producing cells in peripheral blood mononuclear cells (PBMCs) were evaluated at 3 and 6 weeks after immunization. The average number of RBD-P2–specific IFN-γ–producing cells was slightly higher in nG3 than in nG2 at 3 and 6 weeks. As expected, IFN-γ–producing cells in response to the N protein were detected only in nG3, as the animals in this group were immunized with N (Fig. 4D). We also measured the levels of various cytokines in the supernatants of PBMCs from immunized NHPs; IFN-γ, IL-4, IL-5, IL-13, and tumor necrosis factor–α production in PBMCs from nG2 and nG3 was increased compared to those of nG1 (Fig. 4E).

To confirm the protective effect of RBD-P2 + alum (nG2) and RBD-P2/N + alum (nG3), NHPs were challenged with a total of 10 ml of virus [2.6 × 10^6^, 50% tissue culture infectious dose (TCID_50_)/ml] via intratracheal (4 ml), oral (5 ml), conjunctival (0.5 ml), and intranasal (0.5 ml) routes at 9 weeks after the first immunization. Of note, RBD-P2/N + alum–immunized NHPs showed slightly faster SARS-CoV-2 clearance in throat and lung samples than that induced by RBD-P2 + alum, although it was not statistically significant (Fig. 4, F and G). Also, live SARS-CoV-2 particles were not detected in the lungs of either nG2 or nG3 and were barely detectable in the nose and throat in postchallenges (Fig. 4, F and G).

Moreover, the body temperature of immunized NHPs (nG2 and nG3) was not increased compared with that of nG1 after the virus challenge (Fig. 4H). In addition, SARS-CoV-2 genomic RNA (gRNA) was reduced in total lung analyses of nG2 and nG3 compared with that in the lungs of nG1 (fig. S6A). Further, nG3 showed lower gRNA levels than nG2 in throat swabs and some parts of lung tissue (fig. S6B). NHPs did not show any significant weight loss after challenge (fig. S7A). Hematological and biochemical analyses, including absolute lymphocyte counts, revealed no notable changes in the vaccinated groups (nG2 and nG3) compared with those in control NHPs (nG1) (fig. S7B).

## DISCUSSION

Currently, many types of vaccine candidates against SARS-CoV-2, including inactivated, vectored, nucleotide-based, and recombinant, are being tested in preclinical or clinical trials. Most of these candidates involve at least a portion of the spike protein, and it is still unclear whether immunization in combination with the N protein of SARS-CoV-2 would be beneficial for protection against SARS-CoV-2 challenges (*17*–*19*). In this study, we developed a previously unknown subunit vaccine against SARS-CoV-2 that involves the RBD linked to the P2 epitope of tetanus toxoid to enhance immunogenicity. Consistent with the previous finding, we noted higher neutralizing antibody titers against SARS-CoV-2 in rats when immunized with RBD-P2 than that with RBD. In addition, RBD-P2 + alum effectively induced a neutralizing antibody response in NHPs and provided protection against SARS-CoV-2 challenge. Thus, it seems that immunization with RBD-P2 + alum can induce effective and sufficient virus-neutralizing activity and provide protection. However, we could not ascertain whether RBD-P2 + alum induces long-term humoral immunity, as NHPs were challenged with SARS-CoV-2 6 weeks after boosting.

N proteins are considered highly immunogenic, and the gene encoding the N protein is more stable than that encoding the spike protein (*20*). A DNA vaccine encoding SARS-CoV N protein was reported to induce strong N-specific humoral and cellular immune responses in C57BL/6 mice and achieved a significant reduction in viral titers following challenge (*10*). On the contrary, Buchholz *et al.* reported that immunization with the N protein did not induce a neutralizing antibody response and failed to protect against infection in a SARS-CoV hamster model (*21*). To test whether the inclusion of the N protein as a vaccine antigen could generate more balanced immune responses, we compared the immune responses elicited by RBD-P2 and RBD-P2/N immunization in three different animals—mice, rats, and NHPs. Of note, RBD-P2/N + alum–immunized mice exhibited enhanced T cell–mediated immune responses. Immunization with the N protein and RBD-P2 increased CD69 expression in CD4^+^ T cells as well as IFN-γ and IL-4 production in mice. In NHPs, PBMCs in RBD-P2/N + alum–immunized animals produced IFN-γ when they were stimulated with the N protein; however, production of T_H_2-type cytokines (IL-4, IL-5, and IL-13) was very low. Consistent with these findings, the numbers of IFN-γ–producing cells were increased in the RBD-P2/N + alum group. Furthermore, RBD-P2/N + alum–immunized NHPs showed faster SARS-CoV-2 clearance than that of RBD-P2 + alum immunized NHPs in throat swab samples. Thus, increased T cell activation and T_H_1-prone responses may result in faster viral clearance and constrain viral spread.

In addition to inducing T cell responses, we expect that vaccines based on the N protein may provide effective protection against SARS-CoV-2 variants since the N protein is more conserved than the spike protein. Currently, SARS-CoV-2 spike protein variants have emerged, and it was reported that these variants are resistant to the sera of convalescent patients (*22*). Thus, the combination of an RBD-P2/N immunization may be advantageous in overcoming escape mechanisms of SARS-CoV-2 because of gene mutations encoding the spike protein.

There were limitations to this study. We immunized NHPs with a relatively high dose of RBD-P2 (50 μg) and tested the efficacy of the RBD-P2 and N combination only at a 10:1 ratio. Thus, the effectiveness of the N protein could vary depending on the dose of RBD-P2. Further studies are needed to determine the optimal dose or ratio involving the N protein. Currently, we are evaluating the RBD-P2–based subunit vaccine in clinical trials (phase 1, no. NBP2001) because RBD-P2 and RBP-P2/N showed similar protective efficacy in NHPs.

In summary, our data demonstrated that RBD-P2 + alum immunization elicits a robust neutralizing antibody response and provides complete or near-complete elimination of live SARS-CoV-2 virus particles. Moreover, our findings provide insight into the potential use of the N protein in the development of SARS-CoV-2 vaccines.

## MATERIALS AND METHODS

### Recombinant baculovirus production

The Bac-to-Bac expression system (Invitrogen, Waltham, MA, USA; catalog no. 10359-016) was used to produce SARS-CoV-2 RBD-P2 and N proteins. The gene sequences of RBD and N were obtained from the genome sequence of the Wuhan seafood market pneumonia virus, Wuhan-Hu-1. The gene fragments were synthesized at GenScript (Piscataway, NJ, USA) and cloned into the **pFastBac** vector, and the recombinant plasmid was prepared using *Escherichia coli* DH5α cells. The recombinant **pFastBac** plasmid, which served as a donor plasmid, was transformed into *E. coli* DH10Bac competent cells (Gibco, NY, USA; catalog no. 10361012) harboring a shuttle vector (**bacmid**). The recombinant **bacmid** was transfected into insect cells to produce recombinant baculoviruses. For virus production, Sf9 cells (ATCC CRL-1711TM, lot no. 62058094) derived from *Spodoptera frugiperda* were used. Cell culture supernatant was recovered 3 days after transfection to obtain recombinant baculoviruses.

### Production of RBD-P2 and N protein

Sf9 cells were infected with a baculovirus containing the RBD-P2 gene and cultured in a flask. After 6 days, the baculovirus was harvested by centrifugation and filtration through 0.2-μm filters. An aliquot of the clarified baculovirus preparation was used for RBD-P2 antigen production. The titer of the baculovirus was obtained by rapid titration assay. High Five cells were cultured in a 10-liter bioreactor for RBD-P2 antigen production. The Hi-5 cells were infected with baculovirus harboring the RBD-P2 gene at a multiplicity of infection of 1 to 3 and cultured for 3 days. The cells were removed by centrifugation and filtration through 0.2-μm filters. The RBD-P2 antigen was purified using three columns—anion exchange, hydrophobic interaction, and multifunctional resins—from the separated cell culture supernatant. The RBD-P2 antigen was concentrated for formulation using the tangential flow filtration (TFF) system. The purified RBD-P2 antigen was mixed with alum as an adjuvant at a 1:10 ratio. The N antigen was produced using the same baculovirus and insect system. Its blood vessel invasion and upstream processes were the same as those for RBD-P2. The N antigen was purified from the separated cell culture supernatant using an anion exchange column and concentrated for formulation using the TFF system. The formulation for the adjuvant was the same as that for RBD-P2.

### Mouse handling and care

Female BALB/c and C57BL/6 mice aged 8 to 10 weeks were purchased from Dae-Han Bio-Link (Eumseong-Gun, Chungchenonbuk-do, Republic of Korea) or Samtako (Osan-si, Guenggi-do, Republic of Korea). The mice were housed in the animal facility at the Catholic University of Korea under specific pathogen–free conditions at 21° to 22°C and a 12/12-hour light/dark illumination cycle. The animal facility is fully certified by the Korean Association for Laboratory Animals, and the mice were handled in accordance with the protocols approved by the Catholic University of Korea. All experimental procedures performed on mice in this study followed the guidelines of the Institutional Animal Care and Use Committee of the Catholic University of Korea (approval nos. CUK-IBC-2020-001, CUK-IACUC-2020-014, and CUK-IACUC-2020-015).

### Immunization of mice and rats

BALB/C or C57BL/6 mice were intramuscularly injected with 30 μg of RBD-P2 with or without 3 μg of N formulated with 300 μg of alum (Croda, Yorkshire, UK) twice at 3-week intervals. Sera were collected after the mice were euthanized. For the challenge, hACE2 transgenic mice were intramuscularly injected in the upper thigh once with 30 μg of N formulated with 300 μg of alum two times at 0 and 2 weeks. The hACE2 transgenic mice were challenged with the SARS-CoV-2 virus [5 × 10^5^ plaque-forming units (PFU) per mouse] at 3 weeks after the first immunization, and their body weight and survival rate were determined. All challenged mice were maintained in Biosafety Level 3 (BSL-3) facilities at the Korea Zoonosis Research Institute of Jeonbuk National University (JBNU). All mouse experimental procedures conducted in this experiment followed the guidelines of the Institutional Animal Care and Use Committee of JBNU (approval no. JBNU2020-0155).

Rats aged ~8 to 12 weeks were purchased from Orient Bio Inc. (Seongnam, Republic of Korea) and were intramuscularly injected with 30 or 50 μg of RBD-P2 with or without N at a 1:10 ratio. The proteins were combined with 300 or 500 μg of alum (twice at 3-week intervals). Sera were collected from the rats at the indicated time points and at the time of sacrifice for the detection of serum antibodies against RBD-P2 and N.

### NHP care and study design

Nine Cambodian-origin cynomolgus macaques (*Macaca fascicularis*; three males and six females; aged 6 to 7 years) were used in this study. All animals were housed in infrastructure facilities in the Korea National Primate Research Centre (KNPRC) at the Korea Research Institute of Bioscience and Biotechnology (KRIBB), under animal biosecurity level–2 (ABL-2) or ABL-3, as required. Macaques were anesthetized with a combination of ketamine sodium (10 mg/kg) and tiletamine/zolazepam (5 mg/kg) for immunization, viral challenges, swabs, and blood collection.

As illustrated in Fig. 4A, the macaques were randomly assigned to three sex-balanced groups. The animals were intramuscularly immunized in the upper thigh at weeks 0 and 3 with one of the following formulations: (i) 500 μg of alum (Croda), (ii) 50 μg of RBD-P2 with 500 μg of alum, and (iii) 50 μg of RBD-P2 and 5 μg of N with 500 μg of alum. After 6 weeks from the last immunization, all animals were challenged with a total of 10 ml of the SARS-CoV-2 virus (TCID_50_ = 2.6 × 10^6^/ml) via intratracheal (4 ml), oral (5 ml), conjunctival (0.5 ml), and intranasal (0.5 ml) routes. Clinical examinations, swab sampling, and hematological analysis were conducted daily until necropsy. Challenged macaques were monitored for body weight, rectal temperature, and respiration. Nasopharyngeal and oropharyngeal swab samplings were collected in universal viral transport medium at 0, 1, 2, and 3 days postinfection (dpi) for virus quantification. Hematological data were determined on the basis of EDTA-treated whole blood samples using an automatic hematology analyzer (Mindray BC-5000, Nanshan, China).

All macaques were euthanized and necropsied at 3 dpi. Lung samples were collected at necropsy for viral detection and microscopic examination. All procedures were approved by the KRIBB Institutional Animal Care and Use Committee (approval no. KRIBB-AEC-20191). All procedures were performed in a biosafety cabinet class II in the ABL-3 facility at KNPRC, KRIBB (approval no. KRIBB-IBC-20200206).

### SARS-CoV-2 culture

The SARS-CoV-2 virus (BetaCoV/Korea/KCDC03/2020, accession no. 43326 from the National Culture Collection for Pathogens) was propagated in Vero cells in Dulbecco’s modified Eagle’s medium (DMEM; Welgene Inc. Gyeongsan-si, Gyeongsangbuk-do, Republic of Korea) supplemented with 2% fetal bovine serum (FBS), penicillin (10,000 IU/ml), and streptomycin (10,000 IU/ml) in a humidified 5% CO_2_ incubator as described in our previous report (*23*).

### Neutralization assay for NHPs

Vero cells were seeded in a six-well plate and incubated at 37°C and 5% CO_2_ for 16 to 18 hours. Six weeks after the first immunization, plasma samples were inactivated at 56°C for 30 min and 2-fold serially diluted from 20-fold diluted samples. Diluted plasma samples were mixed with SARS-CoV-2 (50 to 100 PFU per well) at 37°C and 5% CO_2_ for 1 hour and then inoculated into Vero cells for 1 hour under the same incubation conditions. After removing the plasma-virus mixture, 1.5% low–melting temperature agarose in DMEM, supplemented with 2% FBS, was added, and the cells were incubated for 3 days. The cells were fixed with 10% neutral formalin solution overnight and stained with 0.1% crystal violet solution. For each dilution, the number of plaques was counted, and the percentage of neutralization in test samples was compared with that in the control.

### Isolation of PBMCs from NHPs

Blood samples were collected in EDTA-containing tubes, 0 and 2 weeks after immunization. PBMCs were isolated by centrifugation at 3500*g* for 20 min with gentle acceleration using Ficoll-Hypaque (Lymphoprep; Axis-Shield, 1114545). Red blood cells in the PBMCs were lysed in Ammonium-Chloride-Potassium (ACK) lysis buffer (Gibco, Gaithersburg, MD, USA) at 20° to 25°C for 5 min. The PBMCs were washed once with flow cytometry staining buffer [1× phosphate-buffered saline (PBS) with 2% FBS] and resuspended in RPMI medium supplemented with 10% FBS and penicillin/streptomycin (1×).

### Human blood samples

In this study, blood and serum samples were collected from 31 convalescent patients with COVID-19 enrolled from Seoul National University Hospital, Seoul Metropolitan Government–Seoul National University Boramae Medical Center, Kyungpook National University Hospital, Chonnam National University Hospital, and Chung-Ang University Hospital. The study was approved by the Institutional Review Boards of the mentioned hospitals (IRB nos. 2042-005-413, CNUH-2020-130, 2020-05-006, and 20–2020-41). Serum samples were heat-inactivated (20 min at 60°C) before using in binding or neutralizing assays.

### Flow cytometry

Mouse splenocytes (1 × 10^6^) were stained in PBS containing 0.5% FBS for flow cytometry. In total, 0.5 μg of Fcγ Receptor blocker (anti-CD16/CD32 antibody) was added 15 min before fluorochrome-conjugated antibody staining. The following antibodies were obtained from Invitrogen (Waltham, MA, USA): anti-mouse CD4, CD8, CD44, CD62L, and CD103. Anti-mouse CD69 antibodies were purchased from BD Bioscience (San Diego, CA, USA). To distinguish live cells from dead cells, the cells were stained with Fixable Viability Dye eFluor 520 (Invitrogen). After washing, the cells were analyzed using a Cytek Aurora Flow Cytometer (Cytek, Fremont, CA, USA), and the results were interpreted using SpectroFlo software (Cytek).

### ELISpot assays

#### ELISpot assay for mice

Splenocytes of immunized mice were stimulated with 500 ng per well of RBD-P2 or 600 ng per well of N peptide mix for 48 hours. IL-4– and IFN-γ–secreting cells were detected using the mouse IL-4 ELISpot^BASIC^ kit and the mouse IFN-γ ELISpot^BASIC^ kit (Mabtech, Nacka Strand, Sweden), respectively. The manufacturer’s protocols were followed for these assays.

#### ELISpot assay for rats

Splenocytes of immunized rats were stimulated with 300 ng per well of RBD-P2 or 300 ng per well of P2 peptides for 60 hours. IFN-γ–secreting cells were detected using a rat IFN-γ ELISpot kit (R&D Systems, Minneapolis, MN, USA) according to the manufacturer’s instructions.

#### ELISpot assay for NHPs

PBMCs of immunized NHPs were stimulated with 300 ng per well of RBD or 300 ng per well of N or 1 μg per well of P2 peptides for 60 hours. IFN-γ–secreting cells were detected using primate IFN-γ ELISpot kit (R&D Systems) according to the manufacturer’s instructions. The resultant spots were counted on a computer-assisted AID EliSpot Reader System (AID, Strasburg, Germany).

### Mouse ELISA

Antigen-specific IgG1 and IgG2c levels in mouse serum were measured using ELISA. First, a 96-well plate (Corning, NY, USA) was coated with either S, N, or RBD-P2 (100 ng/μl) and incubated at 4°C overnight. After incubation, the wells were blocked with 100 μl of blocking buffer (PBS containing 1% bovine serum albumin) at room temperature for 1 hour. Diluted serum samples were added to the wells, and the plates were incubated at room temperature for 2 hours. After incubation, the wells were washed thrice with 200 μl of PBS containing 0.05% Tween 20 (PBS-T). Horseradish peroxidase–conjugated anti-mouse IgG1 (Bethyl Laboratories, Montgomery, TX, USA) and IgG2c (Novus Biologicals, Centennial, CO, USA) antibodies (diluted 1:1000 to 1:10,000 in PBS) were added to the plates, which were then incubated at room temperature for 1 hour. After three washes with PBS-T, tetramethylbenzidine substrate was added, and the plates were further incubated for 15 min before stopping the reaction with 2 N H_2_SO_4_. The optical density at 450 nm was measured using a GloMax Explorer microplate reader (Promega, Seoul, Republic of Korea).

### Plaque reduction neutralization test

Sera of immunized mice, NHPs, and convalescent patients were serially diluted from 1:10 to 1:5120 with serum-free medium. Virus-serum mixtures were prepared by mixing 100 PFU of SARS-CoV-2 with the diluted serum samples, and the mixtures were incubated at 37°C for 1 hour. Vero cells were inoculated with the virus-serum mixtures, and the plates were incubated at 37°C in the presence of 5% CO_2_ for 1 hour. After virus absorption, the agar overlay medium was added, and the plates were incubated at 37°C under 5% CO_2_ for 3 days. The cells were stained with 0.1% crystal violet solution (Sigma-Aldrich, St. Louis, MO, USA), and the plaques were counted. The percentage of neutralization represented the reduction value, which was calculated as the number of plaques in 100 PFU of the virus-infected well per number of plaques in the virus-serum mixture–infected well (×100).

### Viral quantification

The virus in nasopharyngeal and oropharyngeal swab samples and respiratory tissue, including bronchi and six lobes of the lungs, was quantified using reverse transcription quantitative polymerase chain reaction (RT-qPCR), and the TCID_50_ was determined. Briefly, swab samples were centrifuged (1600*g* for 10 min), and the supernatants were filtered through 0.2-μm syringe filters. Lung and bronchus samples were homogenized using a Precellys Homogenizer (Bertin Instruments, Montigny-le-Bretonneux, France) in 10-fold (w/v) sterile PBS (pH 7.4). After centrifugation, the supernatants were directly inoculated into Vero cells and incubated for 3 days at 37°C to calculate the values of TCID_50_/ml. Viral RNA was extracted from the supernatants of swab and tissue samples using a QIAamp Viral RNA Mini Kit (Qiagen, Germantown, MD, USA). RT-qPCR was conducted with a primer/probe set to detect the *ORF1b* gene as described previously (*24*). Viral RNA copy numbers from swab and tissue samples were calculated using the SARS-CoV-2 RNA standard sample, which was run in parallel with the RT-qPCR reaction.

### Holotomography

Holotomography was performed to visualize and acquire images of protein complexes. A holotomography microscope (HT-2H, Tomocube Inc., Daejeon, Republic of Korea), which is a novel optical microscope that uses diffraction tomography to generate three-dimensional (3D) holographic images of label-free live cells, was used for this purpose. Holotomography provides a 3D refractive index distribution of the protein through which several 2D holographic images can be obtained from different angles to generate a 3D tomogram (*25*). The details of holotomography function and property can be found in the literature (*26*). The acquired data were processed and analyzed with Tomostudio software (Tomocube Inc., Daejeon, Republic of Korea). The protein samples, including RBD-P2, RBD-P2/N, RBD-P2 + alum (RBD-P2 in complex with alum), and RBD-P2/N + alum, were analyzed in this study (fig. S3). All samples were prepared at a final concentration of 30 ng/μl. To visualize the samples, a Tomodish (Tomocube Inc., Daejeon, Republic of Korea) was used, and 25 μl of the sample was loaded into the 20 mm–by–20 mm well.

### Dynamic light scattering measurements

RBD-P2 + alum complex, RBD-P2/N + alum complex, and alum alone were prepared at a concentration of 0.1 mg/ml in 0.05 mM phosphate buffer [including 300 mM NaCl (pH 7.0)]. Dynamic light scattering (DLS) was measured on a ZetaSizer Nano ZS90 (Malvern Instrument, Worcs, UK) equipped with a 4-mW He-Ne laser. All the DLS measurements were performed at 4.0° ± 0.1°C and at a scattering angle of 90°. The apparent *z*-average hydrodynamic diameter (*D*_h_) and polydispersity index (PDI; <μ^2^/Γ^2^) were calculated using the Dispersion Technology Software provided by Malvern.

### Histological analysis

Sectioned lung tissue from experimental animals (mG1 to mG3) was submerged in 10% (v/v) neutral-buffered formalin, dehydrated, paraffin-embedded, and sectioned at a 5-μm thickness for histological examination. The histopathologists were blinded to group distribution when this analysis was conducted, and representative histopathological images were obtained and evaluated using Aperio ImageScope version 12.3 (Leica Biosystems Pathology Imaging, Buffalo Grove, IL, USA). The severity of histopathological changes was determined using a five-point scoring system as follows: 0 = no abnormality detected, 1 = minimal, 2 = mild, 3 = moderate, 4 = moderately severe, and 5 = severe. Distribution was recorded as focal, multifocal, and diffused. Recruitment of inflammatory cells into the lungs (bronchi or blood vessels) and morphological alterations of the tissue were assessed after hematoxylin and eosin staining under light microscopy.

### Statistical analysis

Data are expressed as medians ± 95% confidence interval. Statistical analysis was performed using GraphPad Prism. Analysis was performed using a one-way analysis of variance (ANOVA) with Dunnett’s post hoc for multiple group comparisons and unpaired *t* test to compare between two groups. The type of analysis and statistical interpretation of the results are indicated in the figure legends. Differences were considered statistically significant when *P* < 0.05.

## SUPPLEMENTARY MATERIALS

Supplementary material for this article is available at http://advances.sciencemag.org/cgi/content/full/7/22/eabg7156/DC1
